# Early brain iron changes in Parkinson's disease and isolated rapid eye movement sleep behaviour disorder: a four-year longitudinal multimodal quantitative MRI study

**DOI:** 10.1093/braincomms/fcaf212

**Published:** 2025-06-02

**Authors:** Rahul Gaurav, François-Xavier Lejeune, Mathieu D Santin, Romain Valabrègue, Jean-Baptiste Pérot, Nadya Pyatigorskaya, Graziella Mangone, Smaranda Leu-Semenescu, Nicolas Villain, Marie-Odile Habert, Marie Vidailhet, Isabelle Arnulf, Jean-Christophe Corvol, Stéphane Lehéricy

**Affiliations:** Paris Brain Institute–ICM, Sorbonne University, INSERM U1127, CNRS UMR 7225, Pitié-Salpêtrière Hospital, Paris 75013, France; Movement Investigations and Therapeutics Team (MOV’IT), ICM, Paris 75013, France; Center for NeuroImaging Research (CENIR), ICM, Paris 75013, France; Paris Brain Institute–ICM, Sorbonne University, INSERM U1127, CNRS UMR 7225, Pitié-Salpêtrière Hospital, Paris 75013, France; Data Analysis Core (DAC), ICM, Paris 75013, France; Paris Brain Institute–ICM, Sorbonne University, INSERM U1127, CNRS UMR 7225, Pitié-Salpêtrière Hospital, Paris 75013, France; Center for NeuroImaging Research (CENIR), ICM, Paris 75013, France; Paris Brain Institute–ICM, Sorbonne University, INSERM U1127, CNRS UMR 7225, Pitié-Salpêtrière Hospital, Paris 75013, France; Center for NeuroImaging Research (CENIR), ICM, Paris 75013, France; Paris Brain Institute–ICM, Sorbonne University, INSERM U1127, CNRS UMR 7225, Pitié-Salpêtrière Hospital, Paris 75013, France; Movement Investigations and Therapeutics Team (MOV’IT), ICM, Paris 75013, France; Center for NeuroImaging Research (CENIR), ICM, Paris 75013, France; Paris Brain Institute–ICM, Sorbonne University, INSERM U1127, CNRS UMR 7225, Pitié-Salpêtrière Hospital, Paris 75013, France; Movement Investigations and Therapeutics Team (MOV’IT), ICM, Paris 75013, France; Center for NeuroImaging Research (CENIR), ICM, Paris 75013, France; Department of Neuroradiology, Pitié-Salpêtrière Hospital, AP-HP, Paris 75013, France; Paris Brain Institute–ICM, Sorbonne University, INSERM U1127, CNRS UMR 7225, Pitié-Salpêtrière Hospital, Paris 75013, France; INSERM, Clinical Investigation Center for Neurosciences (CIC), Pitié-Salpêtrière Hospital, Paris 75013, France; Sleep Disorders Unit, Pitié-Salpêtrière Hospital, AP-HP, Paris 75013, France; Paris Brain Institute–ICM, Sorbonne University, INSERM U1127, CNRS UMR 7225, Pitié-Salpêtrière Hospital, Paris 75013, France; Department of Neurology, Pitié-Salpêtrière Hospital, AP-HP, Paris 75013, France; Paris Brain Institute–ICM, Sorbonne University, INSERM U1127, CNRS UMR 7225, Pitié-Salpêtrière Hospital, Paris 75013, France; Department of Nuclear Medicine, Pitié-Salpêtrière Hospital, AP-HP, Paris 75013, France; CNRS, INSERM, Laboratoire D’Imagerie Biomédicale, LIB, Sorbonne University, Paris F-75006, France; Paris Brain Institute–ICM, Sorbonne University, INSERM U1127, CNRS UMR 7225, Pitié-Salpêtrière Hospital, Paris 75013, France; Movement Investigations and Therapeutics Team (MOV’IT), ICM, Paris 75013, France; Department of Neurology, Pitié-Salpêtrière Hospital, AP-HP, Paris 75013, France; Paris Brain Institute–ICM, Sorbonne University, INSERM U1127, CNRS UMR 7225, Pitié-Salpêtrière Hospital, Paris 75013, France; Movement Investigations and Therapeutics Team (MOV’IT), ICM, Paris 75013, France; Sleep Disorders Unit, Pitié-Salpêtrière Hospital, AP-HP, Paris 75013, France; Paris Brain Institute–ICM, Sorbonne University, INSERM U1127, CNRS UMR 7225, Pitié-Salpêtrière Hospital, Paris 75013, France; INSERM, Clinical Investigation Center for Neurosciences (CIC), Pitié-Salpêtrière Hospital, Paris 75013, France; Department of Neurology, Pitié-Salpêtrière Hospital, AP-HP, Paris 75013, France; Paris Brain Institute–ICM, Sorbonne University, INSERM U1127, CNRS UMR 7225, Pitié-Salpêtrière Hospital, Paris 75013, France; Movement Investigations and Therapeutics Team (MOV’IT), ICM, Paris 75013, France; Center for NeuroImaging Research (CENIR), ICM, Paris 75013, France; Department of Neuroradiology, Pitié-Salpêtrière Hospital, AP-HP, Paris 75013, France

**Keywords:** Parkinson’s disease, isolated REM sleep behaviour disorder, MRI, quantitative susceptibility mapping, substantia nigra

## Abstract

Parkinson's disease demonstrates increased iron concentration in the substantia nigra (SN). The progression of iron and its interaction with neuromelanin content and dopaminergic dysregulation from prodromal to early-stage Parkinson's disease remain poorly understood. Using quantitative susceptibility mapping (QSM) and R2* relaxation rate, we investigated brain iron changes in patients with isolated rapid eye movement (REM) sleep behaviour disorder and early-stage Parkinson's disease. Subjects were scanned longitudinally at 3.0 Tesla MRI. QSM and R2* values were calculated in the entire SN and its anterior and posterior dorsal and ventral subdivisions. Baseline and longitudinal group differences were tested using analysis of variance of multiple linear regression models controlling for age and sex and linear mixed-effects modelling respectively. We included 44/36/28 healthy volunteers (HVs), 49/20/11 isolated REM sleep behaviour disorder, 127/88/50 Parkinson's disease at first/second/third visit respectively, separated by a 2-year interval. At baseline, there was a significant increase in QSM and R2* values in Parkinson's disease versus HVs in the posteroventral SN only (QSM: +17.6%%; R2*: +7.1%), which did not reach significance in isolated REM sleep behaviour disorder (QSM: +6.9%, R2*: +3.3%). Longitudinally, only posteroventral SN values demonstrated significant effects for Group and Visit using QSM and R2*. Further, the Group-by-Visit interaction was significant only for QSM. The posteroventral SN iron increased with disease duration and was inversely correlated with the changes in nigral neuromelanin content and striatal DaT levels in Parkinson's disease. The posteroventral nigral iron increased with the progression of the disease as well as dopaminergic denervation in Parkinson's disease. QSM was a stronger quantitative longitudinal marker than R2* in detecting regional nigral iron abnormalities as the disease progressed.

## Teaser

In this four-year longitudinal, prospective, quantitative MRI study, Gaurav et al. demonstrated that posteroventral nigral iron accumulation correlates with disease progression and dopaminergic denervation in Parkinson’s disease patients. QSM was a more sensitive and robust marker than R2* for detecting regional nigral iron abnormalities as the disease advanced.

## Graphical Abstract

**Graphical Abstract:**
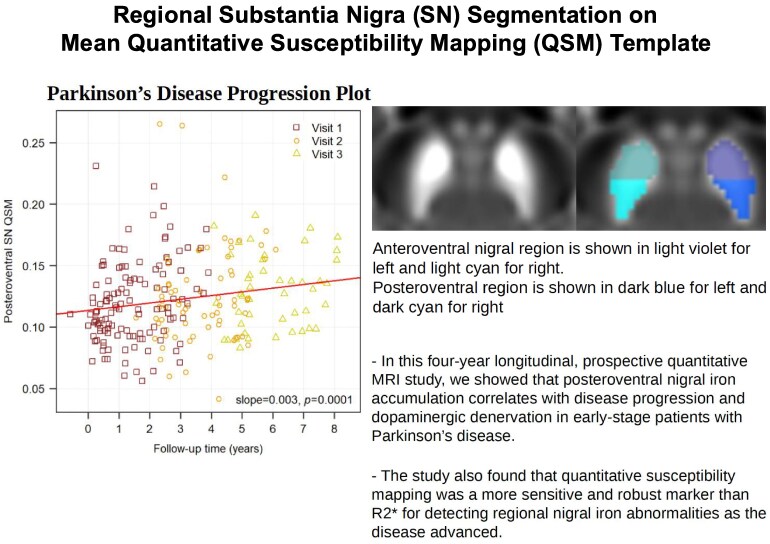

## Introduction

Parkinson's disease (PD) is considered the second most frequent progressive neurodegenerative disorder after Alzheimer's disease affecting 1% of the population above 60 years.^1^ The most frequent neuropathological hallmark of PD is the aggregation of α-synuclein and the progressive depletion of neuromelanin-containing dopaminergic neurons in the substantia nigra (SN) pars compacta (SNc) resulting in a decrease in striatal dopaminergic function.^2^ This is also accompanied by an associated increase in nigral iron concentration.^3^ Iron interactions with dopamine, neuromelanin, and α-synuclein probably contribute to degeneration of dopaminergic nigral neurons.^4^ Iron is mainly stored in the neuromelanin complex of dopaminergic neurons in the SNc.^3^ Excess iron can induce oxidative stress and cell death through the production of toxic dopaminergic derivatives that are ultimately sequestered in the neuromelanin complex.^3^ These alterations occur after an asymptomatic phase, that begins decades before the onset of motor symptoms and can be detected in prodromal parkinsonism such as isolated rapid eye movement (REM) sleep behaviour disorder (RBD).^5^ Iron deposition can be quantified using magnetic resonance imaging (MRI) due to its paramagnetic properties.^6,7^ This results in a reduction of T2* relaxation time or increase in R2* (R2* = 1/T2*), changes in phase in susceptibility-weighted imaging, or elevated susceptibility values on the quantitative susceptibility mapping (QSM). QSM and R2* changes have shown to correlate with iron deposition in post-mortem brains.^8,9^ The major impact of R2* in nigrosome 1, a region of the SNc particularly damaged in PD, derives from the iron accumulated in neuromelanin pigmentation of the dopaminergic neurons.^10^ Some studies using these techniques have reported iron increase in PD in the whole SN,^11-16^ or only in specific parts of the SN comprising the SNc,^7,13,17-21^ the lateral SN^7,22^ or the ventral posterior regions of the SN.^23^ The accuracy of iron measurements to categorize PD patients from healthy volunteers (HVs) are typically in the range of 0.8–0.9.^21,24-26^ Longitudinal studies have shown varying results based on the stage of disease. Many have reported that iron levels increase with the advancement of the disease in the SN in de novo, early and advanced PD using R2* in the entire SN^27^ or both the SN pars reticulata (SNr) and SNc,^28^ only the SNc,^19^ and using QSM in nigrosome 1 in early PD^29^ or the posteroventral SN in moderate PD.^23^ However, a few studies did not find longitudinal iron increase in the whole SN in advanced PD using R2*,^27^ in the SNc or SNr in moderate PD,^30^ or in the dorsal SN adjacent to the red nucleus using QSM.^23^ A reduction in iron content was even reported in the whole SN^31^ and SNr.^19^ Iron increase may begin ∼10 years prior to diagnosis,^17^ and has been observed in de novo and early PD^27,28^ as well as in iRBD^32-34^ although the changes were not significant in some studies using QSM^17,29^ or R2*.^35^ Many studies have reported a positive correlation between iron increase in the SN and the severity of motor changes^27,28,30,36^ and disease duration.^37-39^ Discrepancies between studies could stem from the methodological variations between QSM and R2*, different disease stages ranging from early to advanced, or the specific anatomical focus within the nigral region (territories versus whole). To sum up, while most studies agree to find an increase in iron levels in the SN in PD patients, the longitudinal variations during the disease course and the prodromal stage, the significance of the alterations according to the subregion of the SN or the technique (QSM versus R2*), and the longitudinal variation profile still remains insufficiently characterized.

In this study, we aimed to: (i) gain a more comprehensive understanding of baseline and longitudinal changes in nigral iron concentration from prodromal (iRBD) to early-stage PD (<4 years of disease duration); (ii) compare mean susceptibility values in the entire SN and its territories, and between QSM versus R2*; and (iii) elucidate the association of nigral iron levels with the dopaminergic dysregulation (using [123I] N-ω-fluoropropyl-2β-carbomethoxy-3β-(4-iodophenyl)nortropane (^123^I-FP-CIT) single-photon emission computed tomography (SPECT) imaging), nigral neuromelanin content (using neuromelanin-sensitive MRI) and various clinical examinations.

## Materials and methods

### Participants

This study was part of the ongoing French cohort study called ICEBERG (ClinicalTrials.gov Identifier: NCT02305147). ICEBERG, study was initiated in November 2014 as an observational, prospective, monocentric, longitudinal, case-control study to evaluate clinical features, imaging and biologic biomarkers in patients with PD and the rate of progression compared with the HVs and subjects at risk of developing PD. All subjects in this study underwent motor, neuropsychiatric, sleep, ocular, cognitive evaluations and imaging assessments.

In this study, participants comprising early-stage idiopathic PD, polysomnography-confirmed iRBD and HVs were recruited from May 2015 to March 2024 at the Clinical Investigation Centre of the Paris Brain Institute. The participants were comprehensively clinically assessed three times at first (V1), second (V2) and third visit (V3) separated by an interval of 2.1 ± 0.2 years between V1 and V2 and 2.2 ± 0.4 years between V2 and V3.

The inclusion criteria for patients comprised a clinical diagnosis of PD or iRBD respectively, performed by experienced movement disorder specialists and sleep neurologists, 18–80 years of age, and minimal or no cognitive disturbances (Mini-Mental State Examination score > 26/30) and a disease duration < 4 years for patients with PD, defined as the time elapsed between V1 and the primary manifestation of the motor symptoms. Patients with PD met the UK Parkinson's Disease Society Brain Bank criteria.^40^ The presence of RBD was determined by experienced sleep neurologists using international diagnostic criteria^41^ after a comprehensive interview of the participants or their bed partners and after conducting a video-polysomnography. Patients with iRBD had a history of dream-enacting behaviours with potentially hurtful movements and enhanced tonic chin muscle tone or complex behaviours during REM sleep, but they did not fulfil the criteria for PD or dementia. There were 7 iRBD subjects who converted into overt synucleinopathy over the course of the ICEBERG study. Hence, these converted iRBD subjects were not included in the longitudinal analysis. The HVs did not have any current or prior history of psychiatric or neurological disorders. The local ethics committee approved this study (RCB: 2014-A00725-42) and all participants gave written informed consent.

### Clinical assessments

Clinical examinations included the Hoehn and Yahr scale, which evaluated how the symptoms of the disease progress and the Movement Disorder Society Unified Parkinson's Disease Rating Scale (MDS-UPDRS) part III (OFF and ON condition). MDS-UPDRS part III evaluated motor disability > 12 h after the withdrawal of dopaminergic treatment. The percentage of REM sleep without atonia was calculated from the polysomnographic recordings for PD and iRBD.^42,43^

### Imaging data acquisition

#### Magnetic resonance imaging

All participants were scanned using a 3 Tesla PRISMA MRI system (Siemens Healthineers, Germany) using a 64-channel head coil for signal reception. The MRI protocol included whole brain three-dimensional T_1_-weighted (T_1_-w) imaging and axial turbo spin echo two-dimensional (2D) T_1_-w neuromelanin-sensitive imaging with a field of view restricted to the midbrain. The neuromelanin-sensitive images were carefully positioned in the axial plane perpendicular to the long axis of the brainstem and covering the locus coeruleus/subcoeruleus complex and the SNc. The whole brain T_1_-w images were acquired using a sagittal Magnetisation Prepared 2 RApid Gradient Echo (MP2RAGE) with a 1-mm isovoxel size and the neuromelanin-sensitive images were acquired with the following parameters: with repetition time/echo time/flip angle: 890 ms/13 ms/180°, 3 averages, voxel size: 0.4 × 0.4 × 3 mm^3^, acquisition time: 6:55 min. Whole-brain T_2_*-weighted multi-echo 3D FLASH (fast low angle shot) images were acquired for iron quantification facilitating reconstruction of QSM images. Supplementary Table 1 lists all the parameters for the MRI acquisitions. We carefully inspected the images visually and excluded any with severe motion artefacts, arterial flow artefacts obstructing the visibility of SN, or incomplete coverage of SN.

#### Single-photon emission computed tomography

The dopamine transporter (DaT) single-photon emission computed tomography (SPECT) imaging was carried out on a hybrid gamma camera Discovery 670 Pro system (GE Healthcare) using the [123I] N-ω-fluoropropyl-2β-carbomethoxy-3β-(4-iodophenyl) nortropane (^123^I-FP-CIT) radiotracer. The patients with PD did not disrupt their medications before the SPECT acquisition. The participants were injected with 185 MBq of ^123^I-FP-CIT one hour after the intake of a single dose of Lugol. The acquisition began approximately 180 min after the injection. Further methodological details have been previously described.^17,44^

### Image analyses

Image processing and analysis were conducted using software programs developed using in-house algorithms in MATLAB (The MathWorks Inc., MA, USA, vR2017b) combined with Statistical Parametric Mapping (SPM12, UK), FreeSurfer (MGH, USA, v5.3.0), FSL (FMRIB, UK, v5.0) and NiftyReg (v1.5.58).

#### QSM volume reconstruction

For subject wise QSM calculation, the T_2_* gradient echo signal magnitude and phase from multiple channels of the receive coil were combined using an in-house optimized method as detailed previously.^17^ Then, we computed the local field map using non-linear fitting of the complex gradient echo signal over echo times. Background magnetic fields were subsequently removed using the Laplacian boundary value method. The inverse problem of local field-to-magnetic susceptibility was then solved using the L1-Morphology Enabled Dipole Inversion method.

#### R2* volume reconstruction

R2* maps were estimated by a non-linear fitting of the signal values over echo times. We employed a non-linear regression without water-fat separation using the ‘nlinfit’ function (https://fr.mathworks.com/help/stats/nlinfit.html) of MATLAB (The MathWorks, Inc., MA, USA, vR2017b).

#### Building QSM mean template

We used the aforementioned reconstructed QSM volumes to develop a study-specific mean brain template of QSM using antsBuildtemplate script of advanced normalisation tools (ANTs, http://stnava.github.io/ANTs/) to form a balanced groups representation of 114 subjects altogether at V1 (38 each in HVs, iRBD and PD groups) similar to our previous study.^45^

#### Selection of regional territories on QSM and R2*

Bilateral regions of interest (ROIs)s including the whole SN, subthalamic nucleus (STN), anterior and posterior territories of the dorsal and ventral SN were delineated manually on the aforementioned study-specific QSM template once by an expert rater [RG] using MRView (MRtrix, v3.0.1). No background ROI was delineated. Further, these ROIs on the template were verified and validated by two senior expert raters [NP, SL].

**Figure 1:**
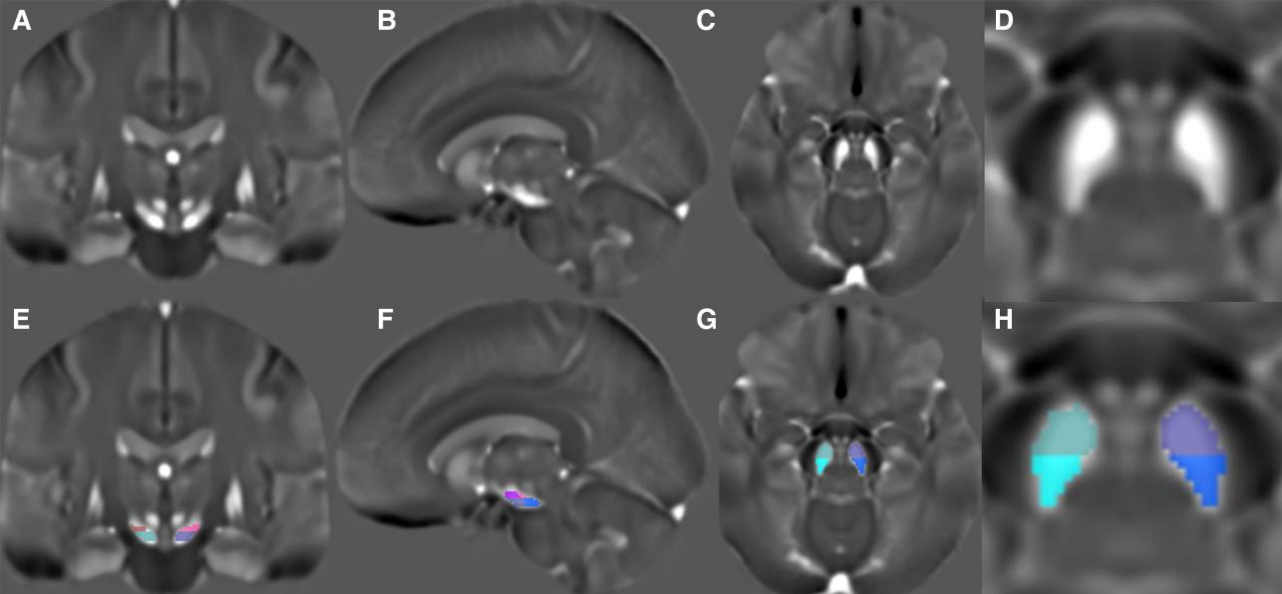
**Brain template.** Brain template image using quantitative susceptibility mapping demonstrating SN without (**A–D**) and with (**E–H**) nigral regions of interest. The panels (**D**) and (**H**) are the zoomed versions of (**C**) and (**G**) respectively. Panel (**E**): posterodorsal SN left is shown in pink and right is shown in dark red, anteroventral left is shown in violet and right is shown in light cyan. Panel (**F**): posterodorsal left is shown in pink, anterodorsal left in magenta, anteroventral left in violet and posteroventral left in dark blue. Panel (**G)**: anteroventral left is shown in violet and right in light cyan, posteroventral left in dark blue and right in dark cyan.

Using the mean QSM template, the bilateral dorsal SN ROIs were delineated manually on every slice where the red nucleus was visible as described in some previous studies.^23,46^ Thereafter, the bilateral ventral SN ROIs were also manually delineated on the template in the inferior slices where the red nucleus was either not visible or unclear.^46^ Further, using the position option of MRView, we split both the dorsal and ventral SN halfway through lengthwise to obtain anterior and posterior dorsal territory (hereafter referred to as anterodorsal and posterodorsal) as well as anterior and posterior ventral territory (hereafter referred to as anteroventral and posteroventral) as shown in Fig. 1.

ANTs provided the subject space-to-template transformation as well as the inverse transformation. Eventually, for each subject, we used the inverse transformation to denormalize all the ROIs defined on the mean template onto the native subject space using WarpImageMultiTransform function of ANTs software.

#### Selection of nigral regional territories on neuromelanin-sensitive imaging

- Whole SNc: We employed a fully automatic procedure to segment whole SNc using the NigraNet framework based on convolutional neural network as described earlier.^47,48^
- Regional SNc: We obtained three regional territories inside the SNc in the form of dorsolateral sensorimotor, dorsomedial limbic and ventral associative using previously developed study-specific mean brain template as explained earlier.^17,45,49^ This template was developed using 114 subjects comprising an equal number of participants in HVs, iRBD and PD and has been tested in various cohort studies.^17,45,49^

The average of left and right side was used in the statistical analyses.

#### Selection of striatal regional territories on DaT-SPECT

We obtained the striatal regions such as the nucleus accumbens and the functional territories in the form of sensorimotor, associative and limbic inside the putamen and the caudate nucleus by coregistering a human basal ganglia template (YeB atlas)^50,51^ to the T_1_-w images for segmentation as detailed in the previous study.^17,44^

#### Quantitative analysis

The quantitative mean susceptibility on QSM in parts per million (ppm) and R2* values in hertz were computed in all the ROIs notably the whole SN and its regional territories (anterodorsal, posterodorsal, anteroventral and posteroventral SN) and STN.

With an aim of investigating the relationship between the values obtained in the posteroventral SN using QSM and R2* and nigral neuromelanin content, we computed the whole SNc volume in mm^3^, corrected SNc volume by total intracranial volume (C_vol_) and normalized signal intensity in regional SNc similar to our previous studies.^52,53^ Further, striatal DaT-SPECT specific binding ratios (SBR) were also computed for correlation analysis with the posteroventral SN values using QSM and R2*.^17,44^

### Statistical analyses

All statistical analyses were conducted using R version 4.3.2 (R Development Core Team, 2023), and plots were generated with the ggplot2 R package (v3.4.4).

#### Percentage Rate of Change in Imaging Measures

Quantitative variables were expressed as mean and standard deviation (SD) and categorical variables as numbers and percentages. The percentage change in mean QSM or R2* values (x̅) from an initial state to a final state was reported as a percentage rate:


$$({\bar{x}}_{\mathrm{final}}-{\bar{x}}_{\mathrm{initial}})/{\bar{x}}_{\mathrm{initial}}\times 100.$$

#### Baseline analyses

One-way analysis of variance (ANOVA) and chi-square tests were used to compare demographic and clinical characteristics of the study groups at baseline.

To compare quantitative imaging-based iron levels using QSM and R2*, we performed cross-sectional comparisons of the groups at baseline using multivariate linear regression models by fitting one model per ROI. Each model was fitted to the quantitative MRI values with respect to the 3-level Group factor (HV, iRBD and PD) with the baseline age and sex as covariates. A significant effect of the group was then assessed based on a *F*-test using the function Anova of the car R package (v3.1-2), followed by post hoc pairwise tests using the emmeans R package (v1.8.9) with Tukey's adjustment for multiple testing.

#### Longitudinal analyses

We designed the longitudinal statistical analyses based on the participants who completed at least the first two visits out of three, each subject thereby providing two or three time points of quantitative MRI values.

The brain iron variations observed across visits were investigated using linear mixed-effects models (LMMs, one model fitted by type of quantitative MRI data). In each model, Group (HV, iRBD and PD), Visit (i.e. the follow-up time at each visit in years) and their interaction term Group × Visit were regarded as fixed effects with age at baseline and sex as covariates, while a random intercept effect was applied on subject identifiers. All LMMs were fitted using the lmer function of the lme4 R package (1.1–35.1). The significance of the main and interaction effects was tested by type II Wald chi-square tests using the function Anova of the car R package. For post hoc comparisons, the time slopes, indicating the yearly evolution of the average quantitative MRI of each group, were estimated and compared using the emtrends function of the emmeans R package.

#### Correlation analyses

Correlation analyses of the posteroventral SN values derived from baseline QSM and R2* maps with the nigral neuromelanin content, DaT-SPECT SBR values and clinical variables (Hoehn and Yahr scale, OFF and ON MDS-UPDRS part III OFF and ON condition, disease duration of PD) were performed using Pearson's correlation coefficient (*r*). Additionally, we also performed a correlation analysis between the posteroventral SN values derived from baseline QSM and R2*. Benjamini-Hochberg false discovery rate correction of the *P*-values was applied within each family of tests. The statistical tests were two-sided and the level of statistical significance was set at *P* or adjusted *P* < 0.05 for all tests.

## Results

### Demographic and clinical characteristics

We included 44/36/28 HV, 49/20/11 iRBD, 127/88/50 PD at V1/V2/V3 respectively, separated by an interval of 2.1 ± 0.2 years between V1 and V2, and 2.2 ± 0.4 years between V2 and V3. Overall, age differed between groups (*P* < 0.001) with iRBD patients being significantly older than the other groups. There was a larger proportion of males in the iRBD group than in the other groups (pairwise chi-square tests with Holm correction, *P* = 0.0044) (Table 1).

**Table 1:** Demographic, clinical characteristics and QSM values within the substantia nigra and STN at V1, V2 and V3

	Visit 1				Visit 2				Visit 3			
	HVs	iRBD	PD	*P*-value^a^	HVs	iRBD	PD	*P*-value^a^	HVs	iRBD	PD	*P*-value^a^
	*n* = 44	*n* = 49	*n* = 127		*n* = 36	*n* = 20	*n* = 88		*n* = 28	*n* = 11	*n* = 50	
Age (years)	62.3 (9.7)	67.7 (5.0)	62.3 (9.0)	*<0.001*	64.3 (9.9)	69.0 (4.8)	64.2 (8.0)	*0*.*003*	68.2 (9.1)	71.8 (4.0)	65.7 (7.4)	*0*.*046*
Male	24 (54.5%)	43 (87.8%)	76 (59.8%)	*<0.001*	19 (52.8%)	15 (75%)	49 (55.7%)	0.228	17 (60.7%)	10 (90.9%)	28 (56%)	0.097
Female	20 (45.5%)	6 (12.2%)	51 (40.2%)		17 (47.2%)	5 (25%)	39 (44.3%)		11 (39.3%)	1 (9.1%)	22 (44%)	
Disease duration (years)	N/A	2.4 (3.6)	1.4 (1.0)	0.114	N/A	4.6 (4.8)	3.7 (1.1)	0.424	N/A	8.8 (6.7)	5.933 (1.223)	0.273
MDS-UPDRS III-ON	N/A	0 (0)	26.2 (7.1)	*<0.001*	N/A	19.7 (17.6)	21.2 (13.7)	0.894	N/A	10.3 (20.5)	21.6 (12.5)	0.352
MDS-UPDRS III-OFF	5.6 (5.4)	10.5 (6.3)	29.5 (7.6)	*<0.001*	5.7 (4.6)	14.0 (9.6)	25.9 (15.4)	*<0.001*	3.3 (4.2)	12.5 (13.2)	24.7 (14.1)	*<0.001*
Hoehn and Yahr	0.068 (0.452)	0.592 (0.864)	1.976 (0.235)	*<0.001*	0 (0)	0.950 (0.887)	1.534 (0.830)	*<0.001*	0.040 (0.200)	0.800 (0.919)	1.581 (0.823)	*<0.001*
Whole Substantia Nigra (SN)	0.118 (0.031)	0.130 (0.041)	0.128 (0.034)	0.179	0.120 (0.032)	0.130 (0.062)	0.132 (0.042)	0.364	0.125 (0.033)	0.128 (0.059)	0.126 (0.037)	0.979
Ventral	0.122 (0.032)	0.134 (0.044)	0.136 (0.038)	0.115	0.125 (0.033)	0.135 (0.066)	0.141 (0.046)	0.232	0.130 (0.035)	0.136 (0.060)	0.141 (0.035)	0.434
Posteroventral	0.103 (0.027)	0.110 (0.038)	0.120 (0.033)	*0.006*	0.105 (0.027)	0.110 (0.052)	0.125 (0.040)	*0.025*	0.108 (0.030)	0.109 (0.048)	0.126 (0.031)	*0.046*
Anteroventral	0.140 (0.037)	0.155 (0.050)	0.150 (0.044)	0.257	0.143 (0.040)	0.157 (0.078)	0.154 (0.052)	0.483	0.150 (0.041)	0.159 (0.070)	0.154 (0.039)	0.898
Dorsal	0.112 (0.032)	0.125 (0.039)	0.118 (0.030)	0.169	0.114 (0.033)	0.123 (0.059)	0.120 (0.037)	0.643	0.118 (0.034)	0.123 (0.048)	0.116 (0.030)	0.829
Posterodorsal	0.097 (0.027)	0.105 (0.037)	0.106 (0.025)	0.243	0.099 (0.026)	0.106 (0.060)	0.108 (0.028)	0.231	0.101 (0.026)	0.099 (0.035)	0.106 (0.025)	0.571
Anterodorsal	0.121 (0.036)	0.137 (0.041)	0.125 (0.035)	0.07	0.123 (0.038)	0.133 (0.060)	0.128 (0.043)	0.726	0.129 (0.039)	0.137 (0.056)	0.122 (0.034)	0.614
Subthalamic nucleus	0.089 (0.025)	0.091 (0.027)	0.093 (0.022)	0.591	0.088 (0.022)	0.087 (0.037)	0.092 (0.023)	0.627	0.090 (0.023)	0.086 (0.028)	0.091 (0.021)	0.822

Post hoc comparisons: iRBD older than PD (V1, V2, V3) and HVs (V1); Males more represented in iRBD compared with HVs and PD (V1); MDS-UPDRS part III OFF: PD > iRBD > HVs (V1), PD > iRBD, HVs (V2, V3); Posteroventral SN higher in PD compared with HVs (V1, V2). Quantitative variables are summarized as mean (standard deviation) and Sex as counts and percentages.

HVs, Healthy Volunteers; iRBD, isolated REM sleep Behaviour Disorder; PD, Parkinson's Disease; MDS-UPDRS, Movement Disorder Society Unified Parkinson's Disease Rating Scale; N/A, not applicable.

^a^ *P*-values were calculated from the following tests as appropriate: one-way analysis of variance test with post hoc Tukey's honest significant difference test, Welch's *t*-test and chi-square test with pairwise comparisons for proportions using Bonferroni's correction. *P*-values in italic indicate significant differences between the groups (*P* < 0.05). No adjustment for covariates was performed.

### Imaging measurements

**Figure 2:**
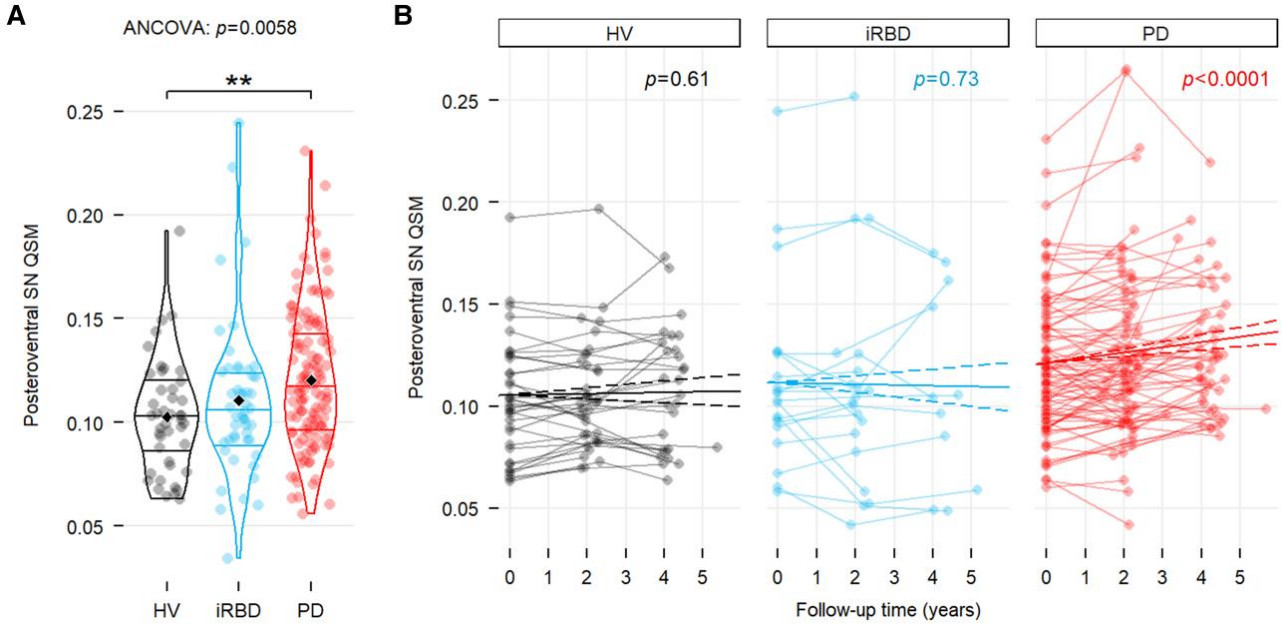
**Baseline and longitudinal plots.** (**A**) Violin plots comparing the baseline distributions of posteroventral SN QSM values with the mean (black diamond point), the median and 25th and 75th percentiles (solid line) between the HVs (black), iRBD (blue) and PD (red) in the posteroventral SN. One-way ANCOVA adjusted for age and sex followed by Tukey's post hoc tests. (**B**) Line plots showing individual longitudinal trajectories for each of the three groups using the QSM images. The bold lines represent the evolution slopes of the change in the groups (solid for the average and dashed for the 95% limits). The estimation of the slopes was obtained based on linear mixed-effects modelling with the R package emmeans and the *P*-value of the test for non-nullity is indicated above the plot. Asterisks: *** *P* < 0.001.

There were no significant differences between the left and right SN QSM values except for a small difference in the posteroventral SN only in the PD group (Wilcoxon signed-rank test: *P* = 0.042). The overall similarity between the left and right values was further supported by strong correlations observed between them (Pearson correlation coefficients ranging from 0.71 to 0.93), justifying their combination. We therefore conducted the imaging analysis by averaging the left and right measurements of the posteroventral SN QSM as well as R2* to reflect the general trend across participants (Table 1, Fig. 2).

#### Baseline analysis

Overall group comparisons demonstrated a significant difference in mean susceptibility values only in the posteroventral SN (QSM: *F*_2,215_ = 5.27, *P* = 0.0058; R2*: *F*_2,208_ = 4.93, *P* = 0.0081, multivariate linear model adjusted for baseline age and sex) and not for any other regions. Using Tukey's post hoc pairwise comparisons, the mean values were significantly increased in PD compared with HVs by +17.6% for QSM (mean difference ± standard error: 0.018 ± 0.006, *P* = 0.0077) and +7.1% for R2* (1.804 ± 0.592 hertz, *P* = 0.0074) but not in iRBD compared with the HVs (+6.9% for QSM: 0.006 ± 0.007, *P* = 0.63; +3.3% for R2*: 0.887 ± 0.743 hertz, *P* = 0.46) or in PD compared with iRBD.

Role of Age and Sex: There was no baseline effect of age in the posteroventral SN (QSM: *F*_1,215_ = 0.016; *P* = 0.90, R2*: *F*_1,208_ = 1.74; *P* = 0.19). There was a significant effect of sex using R2* (QSM: *F*_1,215_ = 0.76; *P* = 0.39, R2*: *F*_1,208_ = 7.97; *P* = 0.0052) with higher values recorded in male subjects (males: 28.5 ± 0.3 hertz; females: 27.1 ± 0.4 hertz).

#### Longitudinal analysis

QSM values demonstrated highly significant Group (type II Wald chi-square test: χ^2^(2) = 8.6, *P* = 0.013), Visit (χ^2^(1) = 17.9, *P* < 0.0001) as well as Group × Visit interaction (χ^2^(2) = 11.2, *P* = 0.0037) effects in the posteroventral SN and not in other regions. The mean slopes indicating the annual change of QSM values in the groups indicated a significant increase in PD patients (slope = 0.0026, 95% CI: 0.0016–0.0035; *P* < 0.0001), but no change in iRBD (slope = −0.00035, 95% CI: −0.0023 to 0.0016; *P* = 0.73) and in HV (slope = 0.0035, 95% CI: −0.0010 to 0.0017; *P* = 0.61). The pairwise comparisons of slopes also demonstrated significant differences for PD with iRBD (*P* = 0.025) and HVs (*P* = 0.022).

The R2* values demonstrated significant Group (χ^2^(2) = 7.8, *P* = 0.021) and Visit (χ^2^(1) = 7.7, *P* = 0.0055) effects in the posteroventral SN but not in other regions, and with no Group × Visit interaction effect (χ^2^(2) = 3.8, *P* = 0.15). The mean slopes of R2* values significantly increased in PD patients (slope = 0.292, 95% CI: 0.120–0.463; *P* = 0.00095), while no change was observed with iRBD (slope = −0.026, 95% CI: −0.378 to 0.326; *P* = 0.89) and HVs (slope = 0.054, 95% CI: −0.225 to 0.333; *P* = 0.70) subjects. However, these slopes were not significantly different from each other (all *P* > 0.24).

**Figure 3:**
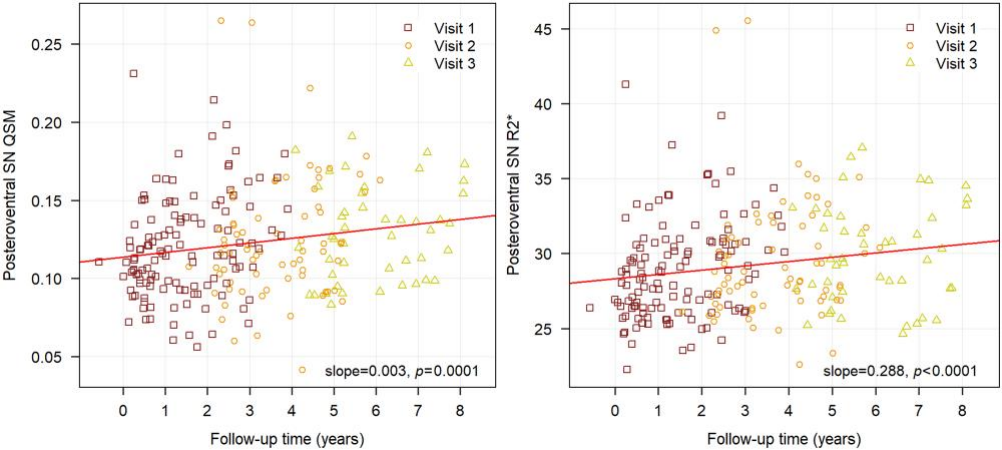
**Progression plot.** PD progression plot of posteroventral SN using QSM and R2* maps.

Rate of Change: During the 4-year follow-up, the mean of QSM values in the posteroventral SN in the PD group increased by 9.2% (mean difference of 0.010 ± 0.002 ppm, *P* < 0.001), while the mean of R2* values increased by 4.2% (mean difference of 1.17 ± 0.35 hertz, *P* = 0.012) (Fig. 3).

Role of Age and Sex: QSM values in the posteroventral SN did not demonstrate any effect of baseline age (*P* = 0.67) or sex (*P* = 0.19). Similar to the baseline results, the R2* values did not demonstrate any effect of baseline age either (*P* = 0.19) but showed a significant effect of sex (*P* = 0.0079) with higher values recorded in male subjects (males: 29.0 ± 0.5 hertz, females: 27.3 ± 0.5 hertz) over the follow-up period.

### Interaction with neuromelanin content

**Figure 4:**
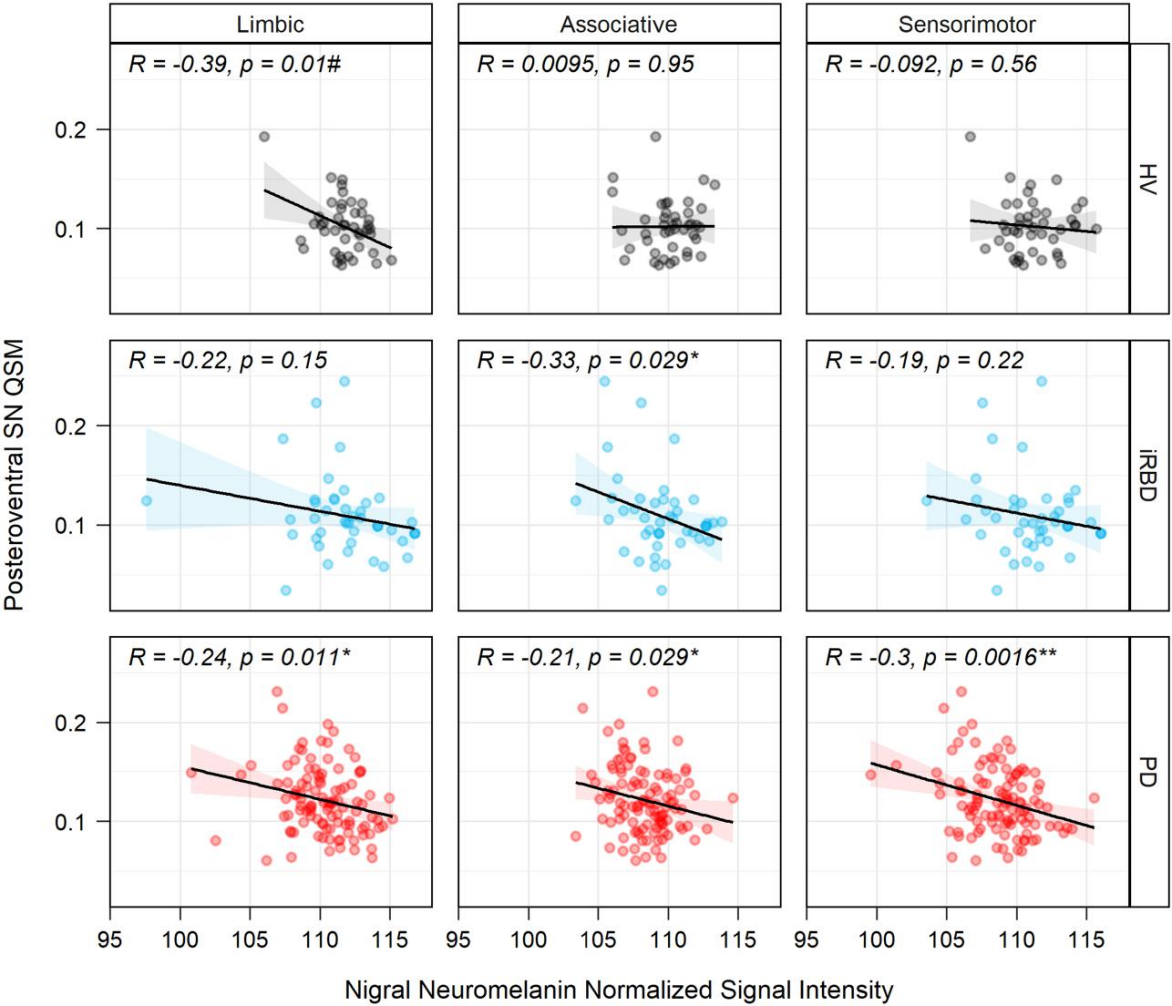
**Scatter plots of posteroventral nigral iron and neuromelanin.** Scatter plots with linear regression line and Pearson's correlation coefficient between posteroventral SN QSM values and neuromelanin content in HVs, iRBD and PD groups (baseline values). Significant correlations were indicated with asterisks: * *P* < 0.05 and ** *P* < 0.01. Further, we added a # sign to emphasize that the association was non-robust as determined by sensitivity analysis after removing a single outlier in limbic subregion of HVs.

For PD patients at baseline, we obtained significantly negative correlations between QSM values in the posteroventral SN and whole SNc volume (*r* = −0.21; *P* = 0.027), corrected whole SNc volume (*r* = −0.25; *P* = 0.007) and normalized nigral neuromelanin signal intensity in limbic (*r* = −0.24; *P* = 0.011), associative (*r* = −0.21; *P* = 0.029) and sensorimotor region (*r* = −0.30; *P* = 0.0016) using neuromelanin-sensitive imaging (Fig. 4). Patients with iRBD only demonstrated significantly negative correlations between QSM values in the posteroventral SN and normalized nigral neuromelanin signal intensity in the associative region (*r* = −0.33; *P* = 0.029). Although we found significant negative correlations between the posteroventral SN QSM values and limbic region in the HVs (*r* = −0.39; *P* = 0.01), sensitivity analysis indicated that this association relied heavily on a single outlier (without outlier, *r* = −0.15, *P* = 0.34). All correlations are presented in Supplementary Fig. 1.

### Interaction with dopaminergic dysfunction

Among the baseline participants, 35 HVs, 37 iRBD and 78 PD had available DaT-SPECT data. Using QSM values in posteroventral SN, PD patients at baseline demonstrated a negative trend with DaT-SBR values in the associative caudate nucleus (*r* = −0.22; *P* = 0.070), limbic caudate nucleus (*r* = −0.21; *P* = 0.091), sensorimotor caudate nucleus (*r* = −0.20; *P* = 0.097) and in limbic putamen (*r* = −0.24; *P* = 0.055). Further, iRBD patients demonstrated negative trend with sensorimotor putamen (*r* = −0.31; *P* = 0.08).

**Figure 5:**
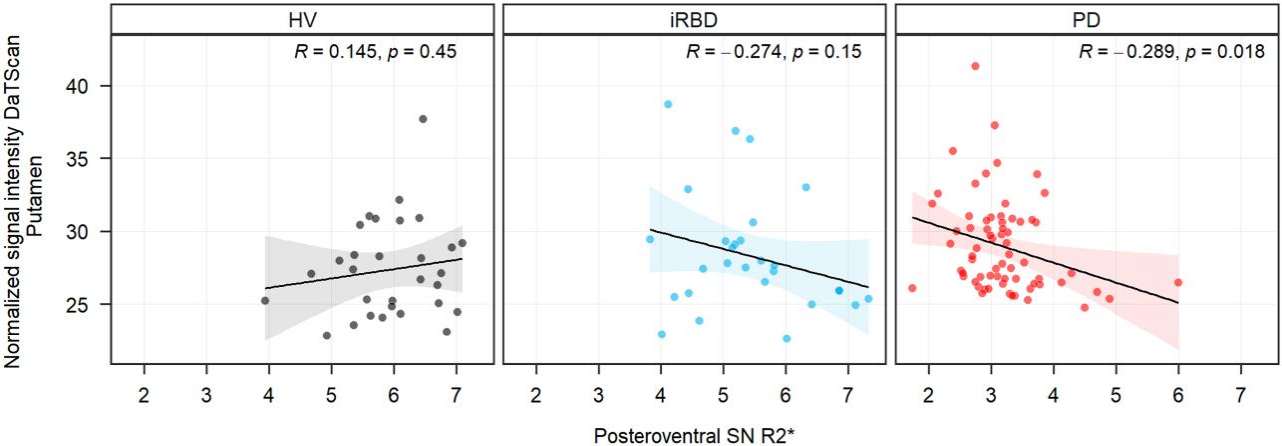
**Scatter plots of posteroventral nigral iron and DaT-SPECT.** Scatter plots with linear regression line and Pearson's correlation coefficient between R2* values in posteroventral SN and DaT-SPECT values in putamen for HVs, iRBD and PD groups (baseline values).

Using R2* values, PD patients demonstrated significantly negative correlations in associative caudate nucleus (*r* = −0.25; *P* = 0.040), limbic caudate nucleus (*r* = −0.27; *P* = 0.030), associative putamen (*r* = −0.26; *P* = 0.033) and limbic putamen (*r* = −0.30; *P* = 0.015, Fig. 5), and negative trend with sensorimotor putamen (*r* = −0.24; *P* = 0.054) and nucleus accumbens (*r* = −0.21; *P* = 0.096). Patients with iRBD showed a negative trend with the sensorimotor putamen (*r* = −0.32; *P* = 0.088). The HVs did not demonstrate any correlations with the DaT-SBR measurements (Supplementary Fig. 2).

### Correlations with clinical Status

For patients with PD at baseline, there was a significant positive correlation between QSM values in the posteroventral SN and disease duration (*r* = 0.31; age-sex adjusted *P* = 0.025). All the other clinical variables did not show any correlation in any group (Supplementary Fig. 3).

### Correlations between QSM and R2*

**Figure 6:**
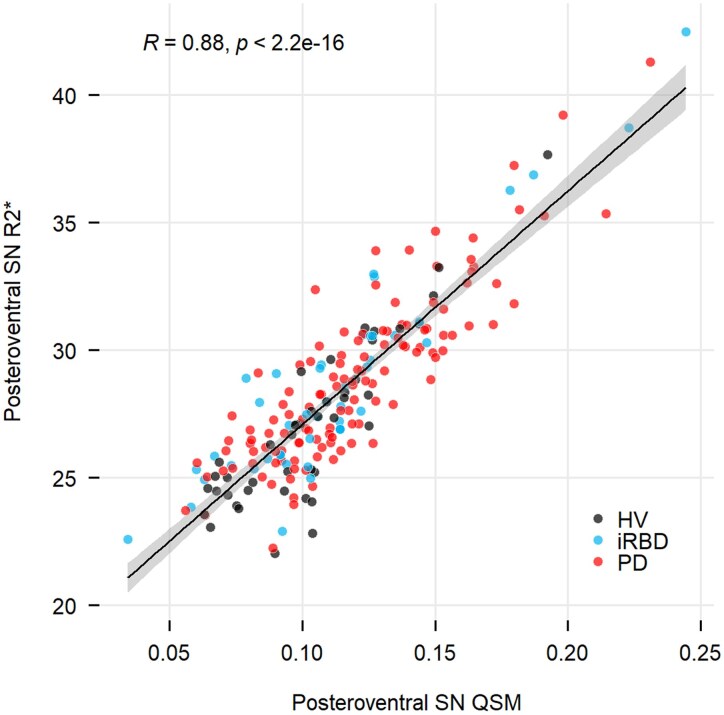
Scatter plots of posteroventral nigral iron using QSM and R2* maps. Scatter plot with linear regression line and Pearson's correlation coefficient between posteroventral SN in QSM and R2* maps.

The posteroventral SN values using QSM showed strong positive correlations with R2* (*r* = 0.88; *P* < 0.0001, Fig. 6).

## Discussion

### Summary

The results demonstrated a highly significant baseline iron increase in both QSM and R2* maps and longitudinal increase in QSM in the posteroventral territory of the SN in patients with early-stage PD. This increase continued to progress as the disease advanced. The values in iRBD patients were consistently intermediate between those of PD patients and HVs, although differences did not reach statistical significance. Furthermore, nigral iron changes in PD patients had a strong relationship with nigral neuromelanin depletion, striatal dopaminergic dysfunction, and disease duration demonstrating the clinical relevance of the imaging measurements. Although globally, QSM values strongly correlated with the R2* values, QSM demonstrated stronger diagnostic performance and longitudinal group wise differences than R2*.

### Quantitative nigral iron changes in Parkinson's disease patients

#### Baseline analysis

We observed a significant baseline iron increase only in the posteroventral SN of PD patients with respect to the HVs. This finding reflected the known pathological pattern of early neurodegeneration taking place in PD patients in the ventral-to-dorsal direction with the progression of PD.^54^ This is also in line with the fact that neurodegeneration in the most dorsal part of the SN, next to the centre of the red nucleus, is mild in PD patients with early and moderate disease duration.^55^ This was also in line with previous MRI studies where iron changes in PD patients were shown to be restricted to the inferior slices where the red nucleus was barely visible corresponding to ventral^23^ or the caudal location.^46^ Although we used template-based segmentation, our SN parcellation was based on ventral-dorsal delineation like a previous study that employed manual segmentation.^23^ The literature on iron-sensitive quantitative imaging techniques has reported variable nigral iron changes. The SN segmentation methods have been shown to vary in previous studies. For instance, some have demonstrated iron augmentation in the whole SN as a single region^12,14,27,56^ unlike our study. Many studies focusing on regional SN have found iron increase in the SNc, which overlapped with the posteroventral SN.^7,13,17-21^ The posteroventral SN was included in the larger entire SN and SNc regions. Our results complemented the previous ones by showing that the increase in iron levels measured in the entire SN or the SNc is mainly driven by the increase in iron levels in the posteroventral part of the SN, which is primarily and more severely affected in PD.

We detected a positive correlation between QSM-derived posteroventral SN values and the mean disease duration of patients with PD. This was in line with several studies with disease duration around 3 years^57^ and 6 years^28,38^ but unlike a study where no correlations were observed.^32^ The correlations with clinical outcomes are often difficult to detect in the early stages of the disease and hence, the literature showed mixed results particularly in studies that included a small number of patients.

#### Longitudinal analysis

We also observed a longitudinal iron increase only in the posteroventral SN of early PD patients of 9.2% using QSM and 4.2% using R2* during the 4-year follow-up with a significant Group × Visit interaction for QSM. This reflected the potential of QSM as a strong disease progression biomarker in detecting nigral iron changes in early PD-stages. These findings were consistent with previous studies where longitudinal iron increase was observed over 3 years using QSM in the ventral posterior SN in PD patients with a disease duration of 6.2 years at baseline,^23^ using R2* in both the SNc and SNr in PD patients with a disease duration of 5.7 years,^28^ and in the whole SN in de novo and early PD patients with a disease duration of 1 to 2 years.^27^ In addition, another longitudinal study reported a Group × Visit interaction effects for R2* in PD patients with varying disease durations.^19^ At later disease stages, iron changes may become more stable^27^or even decrease in the SNr.^19^ Therefore, the changes may differ in later disease stages but were not investigated in the present study. We observed longitudinal Group as well as Visit effects in the posteroventral SN using R2* similar to QSM but the Group × Visit interaction effects did not reach significance even though some studies have reported longitudinal R2* changes in PD.^19,27,28^ Our observations confirm that QSM may be a more effective progression marker than R2* for detecting increased SN iron deposition in line with previous studies.^6,7,13,58^ QSM measures the quantitative susceptibility of local tissues whereas R2* considers both local and surrounding tissue susceptibilities and hence, combines transverse relaxation and local field inhomogeneity.^59^ Nonetheless, both measurements were strongly correlated consistent with previous studies.^19,46^

### Quantitative nigral iron changes in prodromal phase

Patients with iRBD are considered to be in a prodromal stage of synucleinopathies including PD, dementia with Lewy bodies and less frequently multiple system atrophy.^60^ The study of iRBD patients therefore makes it possible to determine how SN iron concentration varies in the prodromal stage of the disease. At baseline, we found a mild increase in nigral iron content in patients with iRBD although not significant, in line with previous studies using QSM^17,29^ and R2*.^35^ As in the present study, QSM values in the nigrosome 1 area in iRBD were intermediate between those of HVs and early PD.^29^ Some other studies have observed a significant nigral iron increase in iRBD.^32,33,61,62^ The variability of results may stem from differences in patient categorisation, the limited size of the sample, the lack of accuracy in determining their precise prodromal stage, and their proximity to conversion into PD and related disorders.^63,64^ In addition, iron changes are known to be slow during this prodromal stage as we did not detect any longitudinal effect in iRBD patients with a shorter follow-up in line with previous studies from our group^17^ or other studies that compared iRBD patients who progressed or not to PD or dementia with Lewy bodies,^29^ although magnetic susceptibility correlated with iRBD duration in another study.^61^

### Effect of sex on brain iron changes

Sex is a much less examined factor in brain iron variability in patients as well as in healthy brains. Here, we did not detect any influence of sex using QSM similar to an age-related brain iron study on healthy adults.^65^ Nevertheless, we detected significant effect of sex using R2* in the posteroventral SN with greater iron levels in male patients and HVs. In general, the literature shows that healthy men demonstrate higher brain iron levels than women.^66-70^ Previous post-mortem study has also shown similar lower overall brain iron levels in females with respect to males.^71^ Various factors such as anemia^72^ and decrease in pre-menstrual blood leading to decrease in peripheral iron levels in females may contribute to sex differences in brain iron storage.^70,73^ Henceforth, sex is a crucial co-factor in studies on brain iron accumulation. This highlights the necessity for future investigation on potential underlying mechanisms that may explain the sex differences in brain iron levels.

### Mechanisms of iron concentration and interaction with neuromelanin and dopamine

Iron plays a crucial role in the neurodegenerative processes in PD.^3^ The upsurge in iron has been associated with reactive oxygen species generation, oxidative stress enhancement in tissues and impairment of cells.^74^ Abnormal iron storage may also contribute to the death of dopaminergic neurons in the SN.^4^ This occurs through various pathways, such as triggering mitochondrial damage leading to the death of cells.^75^ It has been known that the key contribution of R2* in nigrosome 1 comes from the abnormal iron concentration in the melanized dopaminergic neurons.^10^ Here, we confirm that there is a relationship not only between iron deposition and the neuromelanin signal in the SN but also with the striatal dopaminergic dysfunction using DaT-SBR as suggested previously.^17^ With the idea that an iron chelator could restore brain iron levels to normal in PD patients and improve motor impairment, a recent clinical trial demonstrated that although chelators reduced SN iron overload, they exacerbated the symptoms in PD patients.^76^

### Limitations

There were some limitations in this study. Firstly, we used a template-based segmentation method to delineate the nigral territories in QSM and R2* images. This could be upgraded to save time in the future by employing fully automatic convolutional neural network.^77,78^ Secondly, we could not include a sufficient number of iRBD subjects particularly at the last visit. This lack of statistical power could be the reason of lack of significance in the iRBD group. Thirdly, the iRBD group exhibited a high MDS-UPDRS-III OFF score due to due to their proximity to phenoconversion. Fourthly, we did not use a reference region to quantify nigral iron. However, considering the recent guidelines,^79^ a reference region could be useful for multicentric study while controlling for inter-scan variability. Lastly, we must exercise caution in interpreting quantitative MRI-derived brain iron changes. Although iron-sensitive techniques demonstrate magnetic susceptibility changes as iron-based changes, metals like copper and aluminium can also drive the magnetic susceptibility changes, even if this could represent a slight influence.

## Conclusion

This longitudinal study employed both QSM and R2* in a relatively large prospective dataset of prodromal as well as early-stage PD with 2- and 4-year follow-ups. We demonstrated abnormal iron levels during the early stages of PD that increased as the disease progressed and predominated in the SN areas known to be most affected in PD, i.e. the ventral and posterior nigral region, just below the red nucleus level. The changes also showed a strong relationship with the markers of melanized dopaminergic neurons, striatal dopaminergic dysfunction and disease duration. Iron levels were mildly, but not yet significantly, increased in prodromal PD, hence, longer longitudinal studies with a larger sample size are warranted. Further studies combining several neuroimaging biomarkers such as iron, neuromelanin-sensitive and diffusion imaging could furnish complete picture of all potential longitudinal brain changes occurring in prodromal phase to determine and validate the most promising longitudinal biomarkers of dopaminergic dysfunction.

## Supplementary Material

fcaf212_Supplementary_Data

## Data Availability

The data supporting the findings of this study are available from the corresponding author upon reasonable request.
