# Supplementary material for: Early brain iron changes in Parkinson's disease and isolated rapid eye movement sleep behaviour disorder: a four-year longitudinal multimodal quantitative MRI study
Source: Brain Commun. 2025 Jun 2;7(3):fcaf212. doi: 10.1093/braincomms/fcaf212 (PMC12203178; doi:10.1093/braincomms/fcaf212)
Supplement: fcaf212_Supplementary_Data [file fcaf212_supplementary_data.pdf]

	T1-weighted anatomical MRI	T1-weighted neuromelanin-sensitive MRI	T2*-weighted iron sensitive MRI
**Protocol**	3D MP2RAGE	2D TSE	3D FLASH
**Slice Orientation**	Sagittal	Transverse, perpendicular to the longitudinal axis of the brainstem	Transverse
**Echo Time (ms)**	2.98	13	4, 7, 10, 13, 16, 19, 22, 25, 28, 31, 34 and 3
**Repetition Time (ms)**	5000	890	40
**Echo Train Length**	N/A	3	12
**Inversion Time (ms)**	700 and 2500	N/A	N/A
**Flip Angle (degrees)**	4 and 5	90 and 180 (refocus)	20
**Bandwidth (Hertz/pixel)**	240	160	1000
**Field of View: anterior-posterior x right-left x superior-inferior (mm^3)**	256 x 231 x 176	200 x 220 x 48	192 x 174 x 160
**Voxel Size (mm^3)**	1 x 1 x 1	0.43 x 0.43 x 3	1 x 1 x 2<br>reconstructed as 1 isotropic
**Flow Compensation**	N/A	Slice-select direction	First-echo only, all encoding directions
**Acquisition Time (minutes: seconds)**	08:12	06:55	09:18

Supplementary Table 1: Imaging Protocol

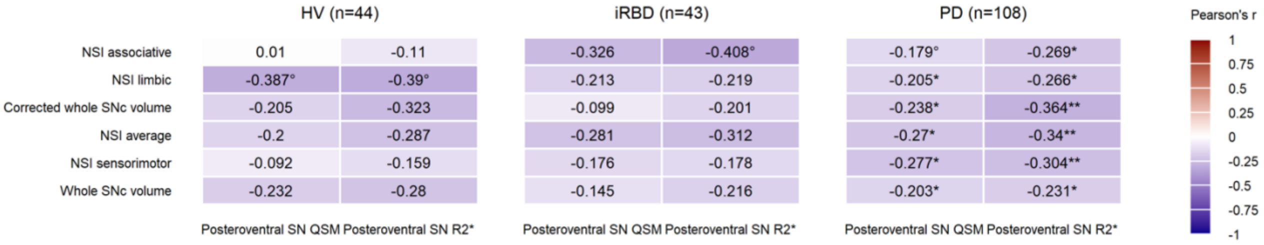

Supplementary Figure 1: Heatmaps between Posteroventral Nigral Iron and Neuromelanin

Heatmaps of Pearson's correlations between posteroventral substantia nigra (SN) and neuromelanin content in healthy volunteers (HV), isolated rapid eye movement sleep behavior disorder (iRBD) and Parkinson's disease (PD) groups (baseline values).

The color scale indicates the strength and direction of the correlation. Red indicates a positive correlation while blue indicates a negative one. °FDR adjusted $p < 0.10$, *FDR adjusted $p < 0.05$, **FDR adjusted $p < 0.01$.

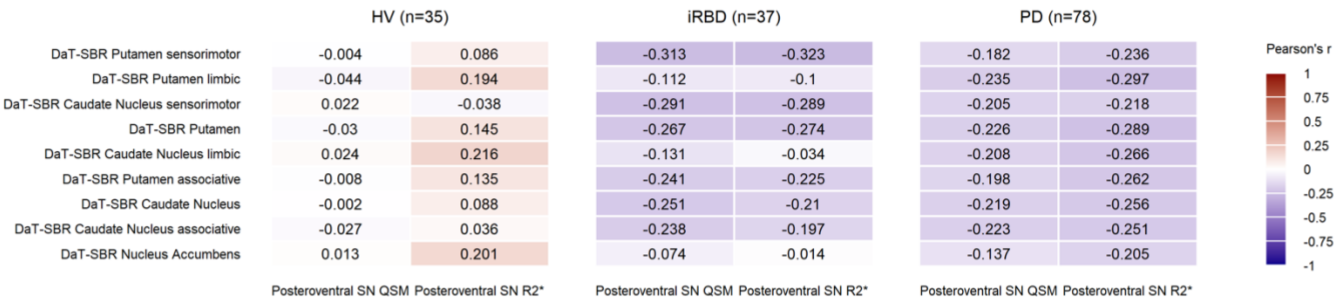

Supplementary Figure 2: Heatmaps Posteroventral Nigral Iron and DaT-SPECT Values

Heatmaps of Pearson's correlations between posteroventral substantia nigra (SN) and DaT-SPECT values in healthy volunteers (HV), isolated rapid eye movement sleep behavior disorder (iRBD) and Parkinson's disease (PD) groups (baseline values). The color scale indicates the strength and direction of the correlation. Red indicates a positive correlation while blue indicates a negative one.

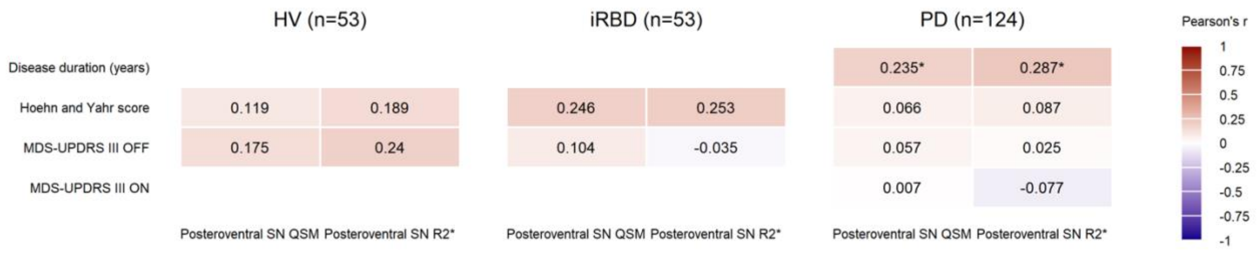

Supplementary Figure 3: Heatmaps between Posteroventral Nigral Iron and Clinical Variables

Heatmaps of Pearson's correlations between posteroventral substantia nigra (SN) and MDS-UPDRS III scores (on and off), Hoehn and Yahr stages and disease duration in healthy volunteers (HV), isolated rapid eye movement sleep behavior disorder (iRBD) and Parkinson's disease (PD) groups (baseline values).

The color scale indicates the strength and direction of the correlation. Red indicates a positive correlation while blue indicates a negative one.

Abbreviation: MDS-UPDRS, Movement Disorder Society Unified Parkinson's Disease Rating Scale.
